# Mitochondrial DNA (mtDNA) Haplogroups Influence the Progression of Knee Osteoarthritis. Data from the Osteoarthritis Initiative (OAI)

**DOI:** 10.1371/journal.pone.0112735

**Published:** 2014-11-12

**Authors:** Angel Soto-Hermida, Mercedes Fernández-Moreno, Natividad Oreiro, Carlos Fernández-López, Sonia Pértega, Estefania Cortés-Pereira, Ignacio Rego-Pérez, Francisco J. Blanco

**Affiliations:** 1 Grupo de Genómica, Servicio de Reumatología, Instituto de Investigación Biomédica de A Coruña (INIBIC), Complexo Hospitalario Universitario de A Coruña (CHUAC), Sergas, Universidade da Coruña (UDC), A Coruña, España; 2 Unidad de Epidemiología. Instituto de Investigacion Biomedica de A Coruña (INIBIC), Complexo Hospitalario Universitario de A Coruña (CHUAC), Sergas, Universidade da Coruña (UDC), A Coruña, España; University of Sevilla, Spain

## Abstract

**Objective:**

To evaluate the influence of the mtDNA haplogroups on knee osteoarthritis progression in Osteoarthritis Initiative (OAI) participants through longitudinal data from radiographs and magnetic resonance imaging (MRI).

**Methods:**

Four-year knee osteoarthritis progression was analyzed as increase in Kellgren and Lawrence (KL) grade, in addition to increase in OARSI atlas grade for joint space narrowing (JSN), osteophytes and subchondral sclerosis in the tibia medial compartment of 891 Caucasian individuals from the progression subcohort. The influence of the haplogroups on the rate of structural progression was also assessed as the four-year change in minimum joint space width (mJSW in millimetres) in both knees of (n = 216) patients with baseline unilateral medial-tibiofemoral JSN. Quantitative cartilage measures from longitudinal MRI data were those related to cartilage thickness and volume with a 24 month follow-up period (n = 381).

**Results:**

During the four-year follow-up period, knee OA patients with the haplogroup T showed the lowest increase in KL grade (Hazard Risk [HR] = 0.499; 95% Confidence Interval [CI]: 0.261–0.819; p<0.05) as well as the lowest cumulative probability of progression for JSN (HR = 0.547; 95% CI: 0.280–0.900; p<0.05), osteophytes (HR = 0.573; 95% CI: 0.304–0.893; p<0.05) and subchondral sclerosis (HR = 0.549; 95% CI: 0.295–0.884; p<0.05). They also showed the lowest decline in mJSW (standardized response means (SRM) = −0.39; p = 0.037) in those knees without baseline medial JSN (no-JSN knees). Normalized cartilage volume loss was significantly lower in patients carrying the haplogroup T at medial tibia femoral (SRM = −0.33; p = 0.023) and central medial femoral (SRM = −0.27; p = 0.031) compartments. Cartilage thickness loss was significantly lower in carriers of haplogroup T at central medial tibia-femoral (SRM = −0.42; p = 0.011), medial tibia femoral (SRM = −0.32; p = 0.018), medial tibia anterior (SRM = +0.31; p = 0.013) and central medial femoral (SRM = −0.19; p = 0.013) compartments.

**Conclusions:**

Mitochondrial genome seems to play a role in the progression of knee osteoarthritis. mtDNA variation could improve identification of patients predisposed to faster or severe progression of the disease.

## Introduction

Osteoarthritis (OA), the most common joint disease related to ageing, is characterized by the degeneration of articular cartilage, affecting subchondral bone and soft tissue and leading to joint destruction and severe impairment of mobility [1]. OA is also the leading cause of permanent work incapacitation and one of the most common reasons for visiting primary care physicians. OA is a multifactorial disease in which a combination of both environmental and genetic factors interact [2]–[4].

At present, therapies available to treat OA are limited. Most current treatments are designed only to relieve pain and reduce or prevent the disability caused by bone and cartilage degeneration. Furthermore, clinical testing of new therapies is complicated by the highly variable way OA manifests in individual patients; it is established that some OA patients remain relatively stable over time, while others progress more rapidly to severe structural deterioration often leading to joint replacement. The usefulness of both genetic and protein biomarkers in OA is to predict, not only the risk of OA at an earlier stage of the disease [5], but also which OA patients are more likely to progress to severe disease. Some studies have evaluated the role of genetic factors in the severity or progression of OA [6]–[9] and, to date, relatively little is known about risk factors for radiographic knee OA progression, despite their great clinical importance.

The Osteoarthritis Initiative (OAI) is a public-private partnership that provides new resources and commitment to help find biochemical, genetic and imaging biomarkers for onset and progression of knee OA. One of the important goals of the OAI is to support development and validation of imaging and biochemical markers that indicate either the presence of OA, or an increased risk for OA, even when radiograph changes are minimal or absent, and which can accurately predict the subsequent course of disease. An essential step to achieve these goals is the assessment of biomarkers in longitudinal studies, over a period of time in which clinical change can be clearly defined, in large, well-characterized populations of persons with OA or who are developing OA.

The OAI cohort study has a public archive of data, biological samples and joint images collected over time from a clinically well characterized population of individuals comprising two subgroups, i) those with clinically significant knee OA who are at risk of disease progression; and ii) individuals who are at high risk for developing clinically significant knee OA. Participants are followed for four years for changes in the clinical status of the knee and other joints, including worsening and onset of symptoms and disabilities, worsening and onset of knee structural abnormalities, and changes in other imaging and biochemical markers of knee OA. Therefore, the use of this cohort of samples confers an extra degree of exceptionality to this work.

In recent years, a growing body of evidence suggests the implication of the mitochondria in the pathogenesis of OA [1], [10]–[12]; mitochondrial function is altered in OA chondrocytes [13] and the mitochondrial dysfunction increases inflammatory responsiveness to cytokines in normal human chondrocytes [14], [15]. Besides, the apoptotic mitochondrial pathway is one of the major cellular pathways for apoptosis of OA chondrocytes [16] and the inhibition of complexes III and V of the mitochondrial respiratory chain (MRC) causes an increased inflammatory response potentially related to the production of reactive oxygen species (ROS) [14]. Mitochondrial free radical production compromises chondrocyte function [17], [18], causing mtDNA damage and reduced mtDNA capacity for repair [19].

In addition to mitochondrial dysfunction, mitochondrial genetics seems to play a role in the OA disease too. Mitochondrial DNA (mtDNA) haplogroups, defined as individual groups characterized by the presence of a particular set of single nucleotide polymorphisms (SNPs) in the mtDNA sequence [20], have been shown to be related to OA at different levels: i) prevalence of the disease in Spanish [6], [21] and Asian [22] populations; ii) complementary genetic markers for the serum levels of collagen type II molecular biomarkers [23] and proteolytic enzymes [24]; and iii) lower nitric oxide (NO) production in human articular chondrocytes as well as higher telomere length [25].

In consideration of this background and the discovery of the role of mitochondria in OA, the aim of this study is to evaluate the occurrence of the mtDNA haplogroups in the progression of knee OA. For this purpose, we used the longitudinal data obtained from radiographs and magnetic resonance images (MRI) of knee OA patients belonging to the progression subcohort of the OAI.

## Materials and Methods

Data used in the preparation of this article were obtained from the Osteoarthritis Initiative (OAI) database, which is available for public access at http://www.oai.ucsf.edu/. The Osteoarthritis Initiative (OAI) is a public-private partnership comprised of five contracts (N01-AR-2-2258; N01-AR-2-2259; N01-AR-2-2260; N01-AR-2-2261; N01-AR-2-2262) funded by the National Institutes of Health, a branch of the Department of Health and Human Services, and conducted by the OAI Study Investigators. Private funding partners include Pfizer, Inc.; Novartis Pharmaceuticals Corporation; Merck Research Laboratories; and GlaxoSmithKline. Private sector funding for the OAI is managed by the Foundation for the National Institutes of Health. The study was reviewed and approved by Comite Etico de Investigación Clínica de Galicia (Ref# 2008/144). Specific datasets used are the 0.2.2 clinical dataset and 0.6, 1.6, 3.5, 5.3, 5.5, 6.3, 0.5 and 1.5 imaging datasets.

### Participants

DNA from all the participants, provided by the OAI, was previously isolated from buffy coat from plasma samples. All the participants analyzed in this study (N = 891) were of Caucasian ancestry and belonged to the progression subcohort of the OAI (**Table 1**). This subcohort includes patients with symptomatic tibiofemoral knee OA at baseline with frequent knee symptoms (pain, aching or stiffness) and radiographic tibiofemoral knee OA defined as tibiofemoral osteophytes (OARSI atlas grades 1–3 [26], equivalent to Kellgren and Lawrence (KL) grade ≥2) on the fixed flexion radiographs.

**Table 1:** Demographic characteristics of the study population at baseline grouped by mitochondrial DNA (mtDNA) haplogroups in the progression subcohort of the OAI.

mtDNA haplogroups							
Characteristic	H (N = 341, 38.27%)	J (N = 89, 9.99%)	T (N = 85, 9.54%)	Uk (N = 228, 25.6%)	Others (N = 148, 16.61%)	p-value	Total (N = 891)
Age at baseline (years)	62.2±9.1	60.3±9.9	61.6±9.5	62.4±9.5	61.4±9.6	0.378*	61.9±9.4
Gender:						0.792^#^	
Male	164 (48.1)	41 (46.1)	45 (52.9)	111 (48.7)	66 (44.6)		427 (47.9)
Female	177 (51.9)	48 (53.9)	40 (47.1)	117 (51.3)	82 (55.4)		464 (52.1)
BMI (Kg/m^2^)	29.6±4.5	29.6±4.8	29.6±4.5	29.2±4.9	29.2±4.4	0.596*	29.4±4.6
BMI≤30	192 (56.3)	50 (56.2)	50 (58.8)	138 (60.5)	92 (62.2)	0.721^#^	522 (58.6)
BMI>30	149 (43.7)	39 (43.8)	35 (41.2)	90 (39.5)	56 (37.8)		369 (41.4)
KL grade at baseline (*worst knee*)**						0.851^#^	
0–I	45 (14.0)	9 (10.8)	9 (11.3)	31 (14.8)	21 (14.9)		115 (13.8)
II–IV	277 (86.0)	74 (89.2)	71 (88.8)	179 (85.2)	120 (85.1)		721 (86.2)
KL grade at baseline (*less severe knee*)						0.538^#^	
0–I	142 (44.1)	39 (47.0)	34 (42.5)	105 (50.0)	71 (50.4)		391 (46.8)
II–IV	180 (55.9)	44 (53.0)	46 (57.5)	105 (50.0)	46 (57.5)		445 (53.2)
mJSN at baseline (*worst knee*)						0.777^#^	
0–I	179 (55.6)	43 (51.8)	46 (57.5)	113 (53.8)	84 (59.6)		465 (55.6)
II–III	143 (44.4)	40 (48.2)	34 (42.5)	97 (46.2)	57 (40.4)		371 (44.4)
mJSN at baseline (*less severe knee*)						0.885^#^	
0–I	280 (87.0)	73 (88.0)	67 (83.8)	180 (85.7)	119 (84.4)		719 (86.0)
II–III	42 (13.0)	10 (12.0)	13 (16.3)	30 (14.3)	22 (15.6)		117 (14.0)
Osteophytes tibia medial (*worst knee*)						0.046^#^	
0–I	192 (67.4)	39 (52.7)	41 (56.9)	107 (57.5)	82 (66.7)		461 (62.3)
II–III	93 (32.6)	35 (47.3)	31 (43.1)	79 (42.5)	41 (33.3)		279 (37.7)
Osteophytes tibia medial (*less severe knee*)						0.577^#^	
0–I	267 (93.7)	68 (91.9)	66 (91.7)	174 (93.5)	119 (96.7)		694 (93.8)
II–III	18 (6.3)	6 (8.1)	6 (8.3)	12 (6.5)	4 (3.3)		46 (6.2)
Sclerosis tibia medial (*worst knee*)						0.131^#^	
0–I	181 (63.5)	37 (50.0)	46 (63.9)	110 (59.1)	83 (67.5)		457 (61.8)
II–III	104 (36.5)	37 (50.0)	26 (36.1)	76 (40.9)	40 (32.5)		283 (38.2)
Sclerosis tibia medial (*less severe knee*)						0.494^#^	
0–I	268 (94.0)	66 (89.2)	65 (90.3)	168 (90.3)	112 (91.1)		679 (91.8)
II–III	17 (6.0)	8 (10.8)	7 (9.7)	18 (9.7)	11 (8.9)		61 (8.2)

Values are mean±standard deviation or number of patients with percentage in parentheses; OAI: osteoarthritis initiative; (*) Kruskal-Wallis non-parametric test for comparison between mtDNA haplogroups; (#) Chi-square test; BMI: body mass index; KL: Kellgren and Lawrence; mJSN: Joint space narrowing in medial compartment; (**) The worst knee and the less severe knee at baseline were designated, respectively, as the knee with the highest and lowest KL (Kellgren andLawrence) grade, OARSI JSN (joint space narrowing) grade, OARSI osteophytes grade or OARSI sclerosis grade, as appropriate on each case; significant p-values are in bold.

The follow-up period was 48 months for radiographic data and 24 months for magnetic resonance imaging (MRI) data.

All the clinical centers of the OAI have made provisions to ensure the safety, confidentiality and ethical treatment of study participants according to the Declaration of Helsinki. In this sense, all the OAI participants signed an informed consent.

### mtDNA haplogroups genotyping

The mtDNA haplogroups of all OA patients were assessed following DNA isolation using a previously described assay [6]. Briefly, a multiplex polymerase chain reaction (PCR) was performed to amplify six mtDNA fragments that contain each of the informative SNPs that characterize the most common European mtDNA haplogroups (H, V, HV*, Uk, J and T). The resulting PCR fragments were further purified and analyzed by the Single Base Extension (SBE) assay and the informative SNPs were visualized after loading the purified SBE product into an ABI 3130 XL genetic analyzer (Applied Biosystems, Foster City, CA, United States). The less common haplogroups (W, I, X and others) were assessed using PCR-restriction fragment length polymorphism (RFLP) according to the hierarchical scheme previously described [6]. The assigned mtDNA haplogroups were verified by sequencing the entire mtDNA control region in 30% of the samples and analysing some of the key SNPs described in phylotree (http://www.phylotree.org).

### Progression criteria

We analyzed radiographic knee OA progression during the follow-up period (48 months) in terms of the KL grade, defining progression as an increase of at least one KL grade in either knee. Additionally, we also analyzed the development or progression of i) joint space narrowing in the medial compartment (mJSN), ii) osteophytes in the tibia medial compartment and iii) subchondral sclerosis in the tibia medial compartment. The progression criterion for each of these three features was an increase of at least one OARSI atlas grade in either knee.

We also analyzed the influence of the mtDNA haplogroups on the rate of structural progression in both knees of patients with baseline unilateral medial JSN (OARSI atlas grade 1–3) and no JSN in the lateral compartment (OARSI atlas grade <1). For this purpose, we analyzed the four-year change in radiographic medial joint space width (mJSW) at the minimum JSW in the medial compartment of both knees (baseline JSN knees and baseline no-JSN knees) of 216 OA patients that met the eligibility criteria.

### Description of the MRI data

For this study, we analyzed the data obtained from measurements of cartilage volume and thickness from serial knee MRI scans performed by Felix Eckstein's group in Germany (Chondrometrics, Gmbh, Ainring) or Austria (Paracelsus University, Salzburg) (http://www.chondrometrics.com/) and contained in different datasets (Projects) of the OAI. These data consisted in a longitudinal study with a 24 month follow-up period of 381 knee OA patients (190 right knees and 191 left knees) in the progression subcohort (namely Project 9). We analyzed data from measurements of the femoral region defined as the 75% of the distance between trochlear notch and the posterior of the femoral condyle (**Figure S1**).

The image analysis relied on sagittal DESS (double echo steady-state) sequence of either the right or left knee [27], [28]. Segmentation of the cartilages was performed at the image analysis center (Chondrometrics GmbH, Ainring, Germany).

### Sample size of the study

The sample size of n = 891 patients allows the probability of OA progression to be estimated with ±3.3% precision using a 95% confidence interval. Additionally, “protective” hazard ratios (HR) < = 0,6 associated with an haplogroup present in at least 10% of the population will be detected as statistically significant with 80% power using a significant level of 0,05. This assumes a censoring probability of 60%.

To analyze the four-year change in medial JSW in patients with unilateral medial JSN, n = 216 OA patients were included in the analysis. Assuming a standard deviation of ±1, this sample size will allow us to detect as statistically significant mean differences of 0.6 or higher for the less frequent haplogroup (n = 24), with 80% power and a significant level of 0.05

For the analysis of cartilage integrity from MRI, data on n = 381 patients are available. This sample size will allow to detect as statistically significant a standardized mean difference of 0,35 or higher in the variables analyzed between the most and the less frequent haplogroup (assuming a ratio 1∶4), with a 95% confidence level and 80% statistical power.

### Statistical analysis

Briefly, for the multivariate analysis of radiographic progression and MRI data, comparisons between haplogroups were performed considering the most common mtDNA haplogroup, H, as the reference group. Therefore, in order to introduce mtDNA haplogroups in the models, a dummy coding was used, with the haplogroup H as the reference group. Since there was no interest in all possible pair-wise comparisons, no additional adjusting for multiple comparisons was done.

Statistical analyses were performed using IBM-SPSS software, release 19 (IBM, Armonk, NY, USA) and R software v3.0.2 (The R Foundation for Statistical Computing). All comparisons were two-sided, with p<0.05 defined as statistically significant.

#### Analysis of radiographic progression

Because the status of a patient was prospectively evaluated at predefined intervals (12, 24, 36 and 48 months), the precise date at which progression occurred could not be determined; it always occurred between visits. Only the interval during which the conversion occurred was observed, therefore such information is said to be interval-censored [29].

To avoid potential biases associated with the use of standard survival analysis in this context, interval-censored data analysis methods were used [29]. Turnbull's extension of the Kaplan-Meier curve to interval-censored data was used to estimate the cumulative probability of progression over time (survivor function) according to the mtDNA haplogroups [30]. An extended Cox proportional hazard model using the iterative convex minorant algorithm was used for multivariate analysis adjusting for the confounder effects of gender, age, BMI, radiographic status of the worst knee (highest KL grade, highest JSN OARSI grade, highest osteophytes OARSI grade or highest sclerosis OARSI grade, as appropriate on each case) and previous surgery, all of them at baseline [31]. Due to difficulties in deriving the asymptotic behavior of statistic tests based on interval-censored data, statistical significance was tested by confidence intervals (CIs) for the hazard ratios (HR) by means of resampling methods. Therefore, CIs were obtained using the bootstrap methodology (1000 replicates) with the percentile method.

To analyze the influence of the mtDNA haplogroups on four-year change in medial JSW (mJSW) from baseline, we focused on patients with unilateral medial JSN and no JSN in the lateral compartment. The JSN knees and no-JSN knees were modeled separately. The mean comparison of the difference between mJSW at baseline and mJSW at final visit (4 years) was further performed by means of an analysis of covariance (ANCOVA) comparing each of the mtDNA haplogroups with the rest pooled together and adjusting for the confounder effects of gender, age and BMI at baseline. Previously, we tested for possible significant associations of the mtDNA haplogroups with KL grade and mJSN at baseline for both JSN and no-JSN knees in this cohort of (n = 216) patients by means of chi-square analyses. Additionally, the standardized response means (SRM), defined as the mean change divided by the standard deviation (SD) of change, was used to measure the sensitivity to change.

#### Analysis of MRI data in the OAI cohort

The parameters analyzed were those related to both cartilage volume and thickness in representative subregions of the joint (**Figure S2**). The parameters for cartilage volume were: cartilage volume in the medial tibia femoral compartment (MFTC.VC) and central medial femoral (cMF.VC) and normalized cartilage volume in the medial tibia femoral compartment (MFTC.VCtAB) and the central medial femoral (cMF.VCtAB). We also analyzed the mean cartilage thickness in the central medial tibia femoral compartment (weight bearing) (cMFTC.ThCtAB), medial tibia femoral compartment (MFTC.ThCtAB), medial tibia (anterior) (aMT.ThCtAB) and central medial femoral (center) (ccMF.ThCtAB).

The mean change (MC) and standard deviation (SD) of change between baseline (T_0_) and 24 months (T_2_) were used as a measure of progression. The SRM was used as a measure of sensitivity to change. The influence of mtDNA haplogroups on changes over time in quantitative MRI scans data, between T_0_ and T_2_, was evaluated though a linear mixed-effects random-intercept and random-slope repeated measures analysis [32]. This model assumes that patient effects (intercepts) and time effects (slopes—MRI data changes over time) are random among patients, taking into account the correlation among repeated measurements in the same individual. Regression coefficients were also estimated for the interaction between time and mtDNA haplogroup, allowing the rate of change to vary from one haplogroup to another. Models were also adjusted for potential confounding factors, including age, gender and BMI.

## Results

### Influence of the mtDNA haplogroups on radiographic knee OA progression

No significant differences in baseline characteristics, including age, gender and BMI of the study population, as well as the radiographic variables at baseline, were detected among the different mtDNA haplogroups, even when analyzed the *worst knee* and the *less severe knee*, except for the presence of osteophytes in the worst knee (p = 0.047) (**Table 1**). The *worst knee* and the *less severe knee* at baseline were designated, respectively, as the knee with the highest and lowest KL grade, OARSI JSN grade, OARSI osteophytes grade or OARSI sclerosis grade, as appropriate on each case. We then analyzed the increase of KL grade in knee OA patients as well as the radiographic progression or development of JSN, osteophytes and subchondral sclerosis.

After adjusting for age, gender, BMI, previous surgery and radiographic status of the worst knee at baseline, the results showed that the percentage of patients that experienced any increase in the KL grade during the follow-up period was significantly lower in carriers of the mtDNA haplogroup T (HR = 0.499; 95% CI: 0.261–0.819; p<0.05); in addition, these patients also showed less development of JSN (HR = 0.547; 95% CI: 0.280–0.900; p<0.05), osteophytes (HR = 0.573; 95% CI: 0.304–0.893; p<0.05) and subchondral sclerosis (HR = 0.549; 95% CI: 0.295–0.884; p<0.05) (**Table 2**).

**Table 2:** Cumulative probability of osteoarthritis progression according to mitochondrial DNA (mtDNA) haplogroups and results of the extended Cox proportional hazard model.

	KL grade			JSN			Osteophytes			Subchondral sclerosis		
Variables	N(%Progressors)^¥^	HR	95% CI^#^	N(%Progressors)^¥^	HR	95% CI^#^	N(%Progressors)^¥^	HR	95% CI^#^	N(%Progressors)^¥^	HR	95% CI^#^
Gender (male)		0.672	0.528–0.859*		1.006	0.755–1.335		0.843	0.650–1.081		1.021	0.791–1.343
Age (years)		1.011	0.999–1.024		0.998	0.982–1.013		0.993	0.980–1.007		1.008	0.994–1.023
BMI (Kg/m^2^)		1.045	1.020–1.069*		1.052	1.021–1.084*		1.028	1.004–1.055*		1.027	0.996–1.056
Previous surgery		0.909	0.702–1.190		0.686	0.487–0.909		0.981	0.739–1.282		1.054	0.788–1.378
Worst knee at bl**^±^**		0.937	0.687–1.305		4.993	3.700–7.373*		1.799	1.405–2.314*		1.396	1.050–1.827*
mtDNA haplogroups												
H (n = 341)	145 (42.5%)	1		111 (32.6%)	1		164 (48.0%)	1		136 (39.8%)	1	
J (n = 89)	40 (44.3%)	1.147	0.731–1.655	28 (31.4%)	0.891	0.581–1.404	45 (51.1%)	1.090	0.715–1.611	29 (32.5%)	0.764	0.487–1.230
Uk (n = 228)	100 (44.1%)	0.986	0.760–1.356	64 (28.4%)	0.741	0.530–1.049	106 (46.8%)	0.906	0.684–1.203	66 (29.1%)	0.632	0.436–0.879
T (n = 85)	19 (22.0%)	0.499	0.261–0.819*	17 (19.7%)	0.547	0.280–0.900*	25 (29.0%)	0.573	0.304–0.893*	21 (25.0%)	0.549	0.295–0.884*
Others (n = 148)	53 (36.0%)	0.786	0.542–1.099	35 (23.7%)	0.699	0.445–1.051	50 (34.1%)	0.687	0.500–1.013	52 (35.1%)	0.847	0.563–1.220

KL: Kellgren and Lawrence; JSN: joint space narrowing; BMI: body mass index; bl: baseline; (**^¥^**): cumulative osteoarthritis progression rate after 48 months of follow-up; HR: hazard ratio; CI: confidence interval; (#): confidence intervals for the hazard ratios obtained using the bootstrap methodology by the percentile method; (±): the worst knee at baseline refers to the radiological status of the knee attending to (highest) KL grade, JSN grade, osteophytes grade or sclerosis grade, as appropriate on each case; (*): statistical significance declared at p≤0.05.

BMI at baseline was found to be a risk factor for radiographic progression (p<0.05) in terms, not only of KL grade (HR = 1.045; 95% CI: 1.020 – 1.069), but also JSN (HR = 1.052; 95% CI: 1.021–1.084) and osteophytes (HR = 1.028; 95% CI: 1.004–1.055) besides, radiographic status of worst knee at baseline significantly associates (p<0.05) with increased development of JSN (HR = 4.993; 95% CI: 3.700–7.373), osteophytes (HR = 1.799; 95% CI: 1.405–2.314) and sclerosis (HR = 1.396; 95% CI: 1.050–1.827) during the follow-up period; male gender was also associated with lower risk for disease progression (p<0.05) in terms of KL grade (HR = 0.672; 95% CI: 0.528–0.859) (**Table 2**).

The analysis of the influence of the mtDNA haplogroups on the rate of structural progression in no-JSN knees, assessed by means of the minimum mJSW, showed carriers of the mtDNA haplogroup T with the lowest decline over time (SRM = −0.39; p = 0.037) (**Table 3**). Additionally, no significant differences were detected in KL grade and mJSN at baseline, in both JSN and no-JSN knees, among the mtDNA haplogroups in this cohort of 216 patients (data not shown).

**Table 3:** Mean differences in mJSW change over 4 years at minimum mJSW among the mitochondrial DNA (mtDNA) haplogroups in 216 OA patients.

	JSN knees (N = 216)			No-JSN knees (N = 216)		
mtDNA haplogroups	Mean change±SD	SRM	p-value^#^	Mean change±SD	SRM	p-value^#^
H (N = 87)	−0.52±0.68	−0.77	0.688	−0.56±1.07	−0.52	0.754
J (N = 24)	−0.75±0.86	−0.87	0.217	−0.62±0.78	−0.79	0.578
T (N = 24)	−0.56±0.77	−0.73	0.919	−0.14±0.37	−0.39	0.034*
Uk (N = 44)	−0.53±0.68	−0.77	0.995	−0.61±1.12	−0.59	0.336
Others (N = 37)	−0.48±0.68	−0.71	0.662	−0.45±0.65	−0.69	0.886

Data are mean±standard deviation, in millimetres (mm); mJSW: medial joint space width; JSN: joint space narrowing; SD: standard deviation; SRM: standardized response means (mean divided by SD); (#) multivariate (ANCOVA) analysis, adjusting for gender, age and body mass index (BMI) at baseline; (*) indicates statistical significance at p≤0.05.

### Influence of the mtDNA haplogroups on knee cartilage integrity. Data from MRI

#### Longitudinal analysis of cartilage volume

**Figure 1:**
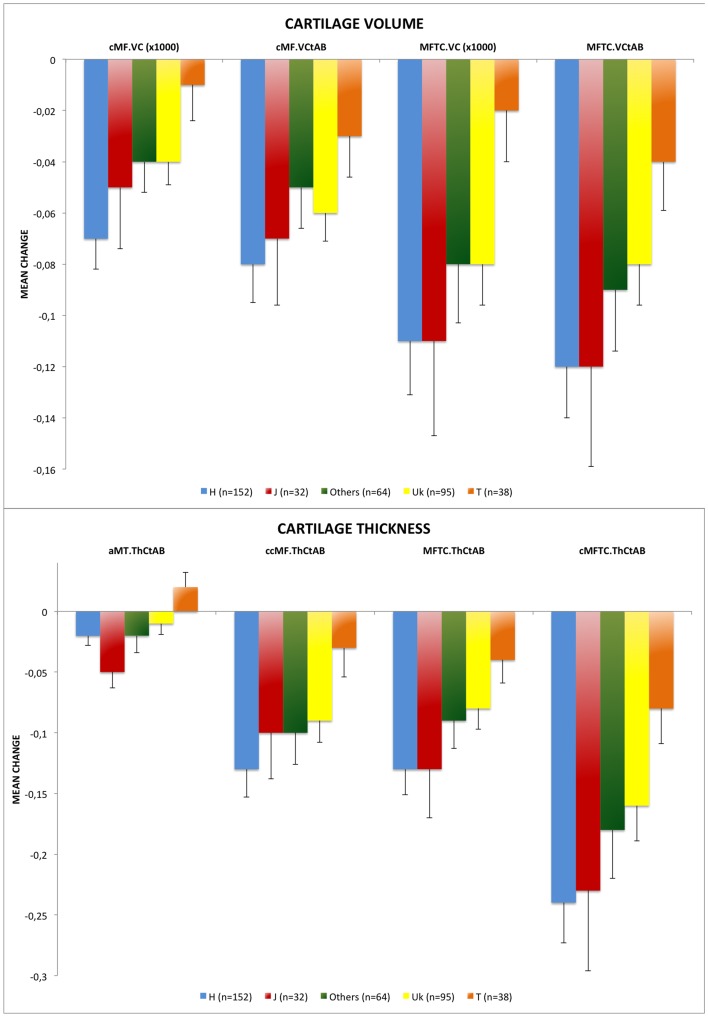
Figure 1a. Longitudinal change between baseline and 24 months in cartilage volume grouped by mitochondrial DNA (mtDNA) haplogroups. MFTC.VCtAB: normalized cartilage volume in medial tibia femoral compartment; cMF.VCtAB: normalized cartilage volume in central medial femoral; MFTC.VC: cartilage volume in medial tibia femoral compartment; cMF.VC: volume of cartilage in central medial femoral. MFTC.VC and cMF.VC that are shown in cubic millimeters (mm3) and MFTC.VCtAB and cMF.VCtAB are shown in square centimeters (cm2); (*) indicates statistical significance (p<0.05). Values are mean change+SE (standard error). **Figure 1b**. Longitudinal change between baseline and 24 months in cartilage thickness grouped by mitochondrial DNA (mtDNA) haplogroups. cMFTC.ThCtAB: mean cartilage thickness in central medial tibia femoral compartment (weight-bearing); MFTC.ThCtAB: mean cartilage thickness in medial tibia femoral compartment; aMT.ThCtAB: mean cartilage thickness in medial tibia (anterior); ccMF.ThCtAB: mean cartilage thickness in central medial femoral (center). All of them represented in millimeters (mm); (*) indicates statistical significance (p<0.05). Values are mean change+SE (standard error).

A significant decline in knee cartilage volume over time was detected (p<0.001). Patients that carry the mtDNA haplogroup T showed significantly lower declines in volume over time in medial tibia femoral (p = 0.015) (MC = −0.02×10^3^±0.12 mm^3^) and central medial femoral (p = 0.015) (MC = −0.01×10^3^±0.08 mm^3^) compartments compared with the most common mtDNA haplogroup H (MC = −0.11×10^3^±0.25 mm^3^ and −0.07×10^3^±0.15 mm^3^, respectively). When the normalized cartilage volume (cartilage volume divided by total area of subchondral bone) of these two subregions was analyzed, the results obtained were similar and carriers of the mtDNA haplogroup T also showed significantly lower declines in normalized volume in medial tibia femoral (p = 0.023) (MC = −0.04±0.12 mm) and central medial femoral (p = 0.031) (MC = −0.03±0.10 mm) compartments (MC = −0.12±0.25 mm and −0.08±0.18 mm, respectively) (**Figure 1a**, **Table 4** and **Table S1**). No differences were found in the rate of change between mtDNA haplogroup H and the other mtDNA haplogroups.

**Table 4:** Random-coefficients linear mixed model to analyze the influence of mtDNA haplogroups on change in cartilage volume (upper) and thickness (lower) over time in 381 knee OA patients in the progression subcohort of the OAI.

	Normalized cartilage volume in medial tibia femoral compartment			Normalized cartilage volume in central medial femoral			Cartilage volume in medial tibia femoral compartment			Cartilage volume in central medial femoral		
	B	SE	p-value^#^	B	SE	p-value^#^	B	SE	p-value^#^	B	SE	p-value^#^
**Age**	−0.011	0.003	0.001*	−0.008	0.002	<0.001*	−10.432	4.044	0.010*	−6.470	2.196	0.003*
**Gender (Female)**	−0.498	0.062	<0.001*	−0.294	0.042	<0.001*	−1211.529	71.933	<0.001*	−510.377	39.069	<0.001*
**BMI**	−0.008	0.006	0.193	−0.008	0.004	0.064	−0.34	7.543	0.964	−0.994	4.097	0.808
**Haplogroup^†^**												
H (n = 152)	0			0			0			0		
J (n = 32)	−0.239	0.120	0.047*	−0.173	0.080	0.032*	−353.483	135.435	0.009*	−191.118	74.039	0.010*
T (n = 38)	0.008	0.112	0.936	0.023	0.075	0.756	−66.358	126.596	0.600	−14.524	69.205	0.840
Uk (n = 95)	0.037	0.080	0.641	0.023	0.054	0.671	8.972	91.019	0.921	−6.14	49.759	0.902
Others (n = 64)	−0.112	0.092	0.222	−0.067	0.062	0.276	−239.768	103.953	0.022*	−120.847	56.828	0.034*
**Time (years)**	−0.061	0.008	<0.001*	−0.042	0.006	<0.001*	−57.915	8.369	<0.001*	−33.201	4.979	<0.001*
**Time x Haplogroup^¥^**												
Time x H	0			0			0			0		
Time x J	0.002	0.020	0.927	0.008	0.014	0.552	1.708	20.069	0.932	5.474	11.940	0.647
Time x T	0.043	0.019	0.023*	0.029	0.013	0.030*	45.808	18.715	0.015*	27.020	11.134	0.015*
Time x Uk	0.021	0.013	0.121	0.013	0.010	0.155	17.160	13.495	0.204	11.489	8.029	0.153
Time x Others	0.016	0.015	0.292	0.015	0.011	0.183	18.899	15.375	0.219	13.807	9.148	0.132

	Mean cartilage thickness in central medial tibia femoral compartment			Mean cartilage thickness in medial tibia femoral compartment			Mean cartilage thickness in medial tibia (anterior)			Mean cartilage thickness in central medial femoral (center)		
	B	SE	p-value^#^	B	SE	p-value^#^	B	SE	p-value^#^	B	SE	p-value^#^
**Age**	−0.012	0.005	0.022*	−0.011	0.003	0.002*	−0.011	0.003	0.002*	−0.007	0.003	0.042*
**Gender (Female)**	−0.641	0.095	<0.001*	−0.481	0.063	<0.001*	−0.481	0.063	<0.001*	−0.377	0.059	<0.001*
**BMI**	−0.018	0.010	0.077	−0.009	0.006	0.180	−0.010	0.006	0.180	−0.013	0.006	0.029
**Haplogroup^†^**												
H (n = 152)	0			0			0			0		
J (n = 32)	−0.265	0.186	0.154	−0.226	0.121	0.062	−0.226	0.121	0.062	−0.188	0.114	0.998
T (n = 38)	0.099	0.174	0.570	0.020	0.113	0.858	0.020	0.112	0.858	0.088	0.107	0.408
Uk (n = 95)	0.102	0.125	0.412	0.044	0.081	0.585	0.044	0.081	0.585	0.041	0.077	0.592
Others (n = 64)	−0.117	0.143	0.413	−0.105	0.093	0.260	−0.105	0.092	0.257	−0.043	0.088	0.623
**Time (years)**	−0.119	0.014	<0.001*	−0.064	0.009	<0.001*	−0.064	0.009	<0.001*	−0.067	0.009	<0.001*
**Time x Haplogroup^¥^**												
Time x H	0			0			0			0		
Time x J	0.006	0.034	0.852	0.001	0.021	0.970	0.001	0.021	0.969	0.018	0.023	0.424
Time x T	0.081	0.032	0.011*	0.045	0.019	0.019*	0.045	0.019	0.018*	0.053	0.021	0.013*
Time x Uk	0.040	0.023	0.076	0.023	0.014	0.104	0.022	0.014	0.104	0.021	0.015	0.156
Time x Others	0.028	0.026	0.285	0.020	0.016	0.204	0.020	0.016	0.204	0.017	0.017	0.323

BMI: body mass index; B: regression coefficient; SE: standard error; (†) the coefficient of each haplogroup represents the mean difference between a particular haplogroup and the reference haplogroup H at baseline; (**¥**) shows the interaction between the mtDNA haplogroups and the loss rate of cartilage volume and thickness over time; (#) linear mixed-effects random-intercept and random-slope repeated measures analysis adjusting for gender, age and BMI at baseline and considering the most common mtDNA haplogroup, H, as the reference group; (*): indicates statistical significance at p≤0.05.

#### Longitudinal analysis of cartilage thickness

A significant decline in cartilage thickness over time was observed (p<0.001). When compared with carriers of mtDNA haplogroup H, patients with haplogroup T had significantly lower declines in thickness over time in central medial tibia femoral (weight bearing) (MC = −0.08±0.18 mm *versus* −0.24±0.41 mm, p = 0.011), medial tibia femoral (MC = −0.04±0.12 mm *versus* −0.13±0.26 mm, p = 0.018), medial tibia (anterior) (MC = +0.02±0.08 mm *versus* −0.02±0.10 mm, p = 0.013) and central medial femoral (center) (MC = −0.03±0.14 mm *versus* −0.13±0.29 mm, p = 0.013) compartments (**Figure 1b**, **Table 4** and **Table S1**). Similar to cartilage volume, no differences in the loss rate of knee cartilage thickness over time were detected between mtDNA haplogroup H and the other haplogroups.

## Discussion

To our knowledge, this is the first study to use the well characterized follow-up OAI cohort to analyze the influence of genetic factors on disease progression. Specifically, this study attempts to analyze the influence of mtDNA haplogroups on the progression of knee OA. The results obtained reveal that patients in the progression subcohort carrying mtDNA haplogroup T show not only a significantly lower cumulative probability of progression in KL grade, but also less development of mJSN, osteophytes and subchondral sclerosis in the medial tibia. These observations were further strengthened by analyzing the evolution of cartilage integrity over two years showing a significantly reduced loss of knee cartilage thickness and volume in carriers of mtDNA haplogroup T too.

Previous findings reported in a Spanish cohort showed that patients with haplogroups belonging to cluster TJ had slower radiographic OA progression attending to KL grade [33]. Interestingly, an association study between haplogroups and OA prevalence in Spanish populations revealed a significant overrepresentation of the cluster TJ in radiological knee healthy subjects [6]. This association was further partially replicated in a larger cohort of hip OA patients from the same location, showing an association of the mtDNA haplogroup J with lower prevalence of hip OA [21]. Moreover, in another recent work, the mtDNA haplogroup T appeared overrepresented in knee healthy subjects from the United Kingdom after performing a regression model adjusting for gender and age [34].

To date, the study presented here is the first and most complete progression study involving the mtDNA haplogroups and OA using the cohort of the OAI; the results obtained should not be interpreted as discrepant in relation to previous findings, not only because this work does not analyze the association of mtDNA haplogroups with the prevalence of OA, but also because both above-mentioned mtDNA haplogroups T and J are considered “sister” haplogroups that share the same phylogenetic origin (the mtDNA cluster TJ) [35], [36] that comprises a set of uncoupling mutations which, in combination with certain nuclear backgrounds, have been described to have common functional characteristics in their OXHPOS system such as decreased ATP production and reduced ROS generation [37]–[39].

A recent work by Hudson and co-workers found no evidence of an association between mtDNA variants and the prevalence of OA [40]. However, some points could explain this; first, as postulated by the authors, the relative contribution of specific mtDNA variants could vary in different ethnic groups by means of homoplasy and/or geographic differences in the finer details of sub-haplogroup structures of mtDNA [40]. In this sense, population-specific associations of the mtDNA haplogroups, probably due to their interaction with environmental factors [41] as a result of their adaptation to colder climates [35], [36], [42], have also been described [43], [44]; second, as the authors describe in their manuscript, the GWAS study performed by the arcOGEN consortium also failed to replicate previous associations involving other genes described in OA at genome-wide significance levels, such as GDF5, chromosome 7q22 and MCF2L [45]; third, control samples from the arcOGEN study are only asymptomatic with no radiographic information, thus the selection of adequate healthy controls for association studies in the OA disease is a crucial for successfully conclusions.

The risk of structural progression in a knee without baseline JSN (no-JSN knee) is higher when the contralateral knee has already lost some space (JSN-knee); therefore, some authors suggest that the no-JSN knee would be the target knee in clinical trials because it would be expected to have less damage than a JSN knee but may have a significant progression over time due to the prevalence of contralateral JSN [46], [47]. In order to evaluate the feasibility of detecting drug effects in clinical trials, we have quantified the mJSW following a similar approach to that described by Benichou and co-workers [48]. Results showed that OA patients with the mtDNA haplogroup T had lower rate of structural progression in no-JSN knees. These results point to take into consideration the mtDNA haplogroups when clinical trials in this population are performed.

The medial compartment is generally heavily loaded and knee OA affects this compartment more often than the lateral one [49], [50]; therefore the analysis of MRI images carried out in this work was performed in regions/subregions that were previously characterized among the most sensitive to change in knee OA: the central medial tibia femoral compartment and medial tibia femoral compartment [28], [51]–[54], as well as other regions of interest such as the central medial femoral condyle and medial tibia (anterior). The conclusion drawn is that OA patients carrying the mtDNA haplogroup T had a significantly lower decline of cartilage thickness and volume over time in these medial subregions.

A key part of the variation in different clinical forms of OA is attributable to genetics [55]. In relation to OA progression, previous studies by other investigators reported that genetic variants of genes, such as interleukin-1 receptor antagonist (IL1RN) [9], [56], [57] or cartilage intermediate-layer protein (CILP) [7], influence the severity and progression of OA. A functional polymorphism in the 5′-UTR of the growth differentiation factor-5 (GDF-5) gene has also been consistently associated with OA susceptibility and was part of a prediction model for knee OA based on genetic and clinical information [58], [59]. The results of the present study strengthen the role of genetic variation, including mitochondrial genetics, in OA as previously described [1], [3].

This study has some points that must be clarified; i) in relation to confounding factors, the analyses were finally adjusted for potential confounders such as age, gender, BMI and, in the radiographic progression, previous surgery and the radiographic status of the worst knee at baseline. Other predictors of OA occurrence or progression over time, such as bone marrow lesions or meniscal lesions, were also analyzed but finally not taken into account because of the small number of patients without missing data. On the other hand, ii) a key strength of this study is the analysis performed; the use of interval-censored data analysis to determine the probability of knee OA progression according to the mtDNA haplogroups avoids biases associated with the use of standard survival analysis in this context. Moreover, the use of linear mixed models in the analysis of MRI data allows us to take into account all available observations for each patient over time, therefore providing more accurate results and higher statistical power.

In summary, the results obtained in this work are of special interest because they show not only significant smaller longitudinal radiographic changes in patients carrying the mtDNA haplogroup T, but also significant lower decline in thickness and volume over time in weight bearing cartilage. A possible physiological explanation for these results, in line with what it was described above, is the mtDNA haplogroup T shows higher capacity to cope with oxidative stress than haplogroup H do [37] and oxidative stress is involved in the pathogenesis of OA [60]. This differential behavior is probably related with the origin of the mtDNA haplogroups, which are the result of a process of adaptive selection that permitted humans to adapt to colder climates when emigrated out from Africa [35], [36]. Based on this theory, some of these mtDNA variants, specifically the mtDNA haplogroup H, are highly efficient in transforming the dietary calories into ATP, generating minimum heat and increased ROS; meanwhile, other mtDNA variants, such as mtDNA haplogroups T and J, are less efficient in converting dietary calories into ATP, therefore producing more heat and less ROS [61]. However, these mtDNA variants that have been critical for human adaptation to different global environments (not only temperature, but also changes in food or caloric supply, seasonal variation in climate or even infections) and would have favored survival and reproduction of populations residing in a particular climate zone might be maladaptive in a different environment with new lifestyles [62]. Hence, mtDNA haplogroups have been correlated with predisposition to a wide range of metabolic and degenerative diseases, obesity, cancers and longevity in a population-specific manner [42], [63], [64].

In conclusion, the results obtained in this work point to a possible role of mtDNA variation in the radiographic progression of OA and could improve identification of patients predisposed to faster or more severe progression of the disease. If further validated in additional prospective and well-characterized cohorts, the inclusion of mtDNA haplogroup assignment may also be useful for clinical trials.

## Supporting Information

Figure S1 Sagittal RM images (DESSwe sequence) with the cartilage of MF being divided into cMF and pMF at 60% (left) and 75% (right) of the distance between the trochlear notch and the posterior end of the femoral condyle; MT: medial tibia; MF: medial femoral condyle; cMF: central (weight bearing) medial femoral condyle; pMF: posterior medial femoral condyle.
(TIF)

Figure S2 **Representative subregions of the knee used to track changes in cartilage thickness and volume.** cMF: central (weight-bearing) medial femoral condyle; MT: medial tibia; cLF: central (weight-bearing) lateral femoral condyle; LT: lateral tibia; ccMF: central subregion of central (weight-bearing) medial femur; cMFTC: central medial femoro-tibial compartment; cMT: central subregion of medial tibia; aMT: anterior subregion of medial tibia.
(TIF)

Table S1 Longitudinal change between baseline (T0) and 24 months (T2) in quantitative parameters of cartilage integrity (volume and thickness) grouped by mitochondrial DNA (mtDNA) haplogroups.
(DOCX)

Table S2 Cross-sectional differences among the mitochondrial DNA (mtDNA) haplogroups in quantitative parameters of cartilage structure collected in an additional and different cohort of (n = 326) knee OA patients in the progression subcohort of the OAI with no follow-up (namely Project 18).
(DOCX)
