# Supplementary figures and images for: Mitochondrial DNA (mtDNA) Haplogroups Influence the Progression of Knee Osteoarthritis. Data from the Osteoarthritis Initiative (OAI)
Source: PLoS One. 2014 Nov 12;9(11):e112735. doi: 10.1371/journal.pone.0112735 (PMC4229258; doi:10.1371/journal.pone.0112735)

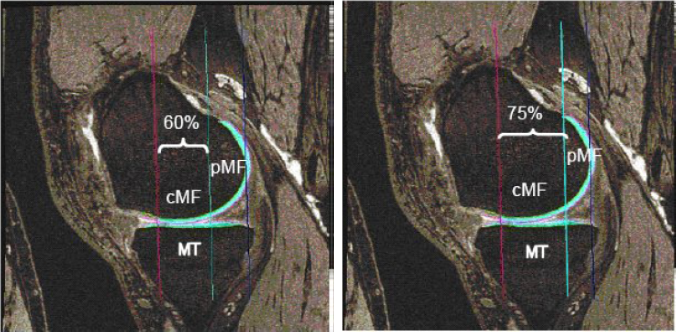

Supplement: Figure S1 — Sagittal RM images (DESSwe sequence) with the cartilage of MF being divided into cMF and pMF at 60% (left) and 75% (right) of the distance between the trochlear notch and the posterior end of the femoral condyle; MT: medial tibia; MF: medial femoral condyle; cMF: central (weight bearing) medial femoral condyle; pMF: posterior medial femoral condyle. (TIF) [file pone.0112735.s001.tif]

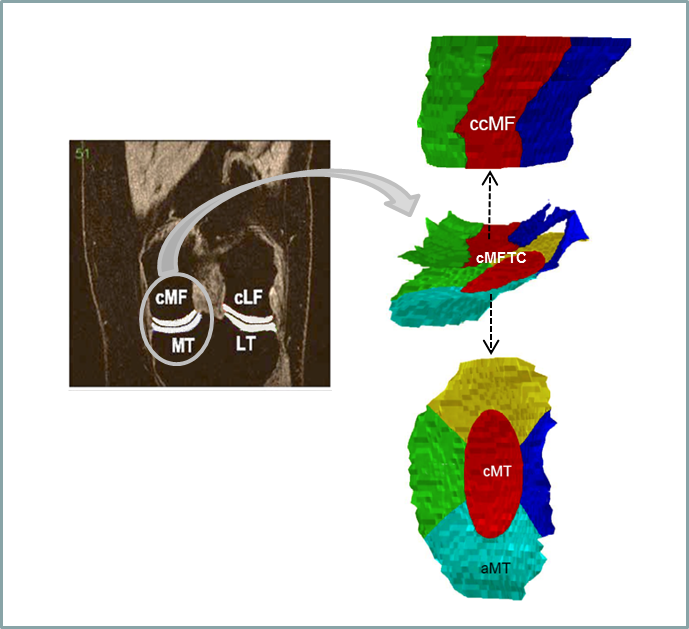

Supplement: Figure S2 — Representative subregions of the knee used to track changes in cartilage thickness and volume. cMF: central (weight-bearing) medial femoral condyle; MT: medial tibia; cLF: central (weight-bearing) lateral femoral condyle; LT: lateral tibia; ccMF: central subregion of central (weight-bearing) medial femur; cMFTC: central medial femoro-tibial compartment; cMT: central subregion of medial tibia; aMT: anterior subregion of medial tibia. (TIF) [file pone.0112735.s002.tif]
