# Supplementary material for: Mitochondrial DNA (mtDNA) Haplogroups Influence the Progression of Knee Osteoarthritis. Data from the Osteoarthritis Initiative (OAI)
Source: PLoS One. 2014 Nov 12;9(11):e112735. doi: 10.1371/journal.pone.0112735 (PMC4229258; doi:10.1371/journal.pone.0112735)
Supplement: Table S1 — Longitudinal change between baseline (T0) and 24 months (T2) in quantitative parameters of cartilage integrity (volume and thickness) grouped by mitochondrial DNA (mtDNA) haplogroups. (DOCX) [file pone.0112735.s003.docx]

| **Table S1.** Longitudinal change between baseline (T_0_) and 24 months (T_2_) in quantitative parameters of cartilage integrity (volume and thickness) grouped by mitochondrial DNA (mtDNA) haplogroups | | | | | | | | | | | |  |
| --- | --- | --- | --- | --- | --- | --- | --- | --- | --- | --- | --- | --- |
|  | **H (n=152)** | | **J (n=32)** | | **T (n=38)** | | **Uk (n=95)** | | | **Others (n=64)** | | |
|  | **Mean change±SD** | **SRM** | **Mean change±SD** | **SRM** | **Mean change±SD** | **SRM** | **Mean change±SD** | **SRM** | **Mean change±SD** | | **SRM** | |
| *Cartilage volume* |  |  |  |  |  |  |  |  |  | |  | |
| MFTC.VCtAB | -0.12±0.25 | -0.50 | -0.12±0.22 | -0.54 | -0.04±0.12 | -0.33 | -0.08±0.16 | -0.51 | -0.09±0.19 | | -0.48 | |
| cMF.VCtAB | -0.08±0.18 | -0.46 | -0.07±0.15 | -0.44 | -0.03±0.10 | -0.27 | -0.06±0.11 | -0.51 | -0.05±0.12 | | -0.44 | |
| MFTC.VC (x1000) | -0.11±0.25 | -0.45 | -0.11±0.21 | -0.53 | -0.02±0.12 | -0.19 | -0.08±0.15 | -0.53 | -0.08±0.18 | | -0.42 | |
| cMF.VC (x1000) | -0.07±0.15 | -0.44 | -0.05±0.13 | -0.41 | -0.01±0.08 | -0.14 | -0.04±0.09 | -0.49 | -0.04±0.10 | | -0.39 | |
| *Cartilage thickness* |  |  |  |  |  |  |  |  |  | |  | |
| cMFTC.ThCtAB | -0.24±0.41 | -0.57 | -0.23±0.38 | -0.60 | -0.08±0.18 | -0.42 | -0.16±0.28 | -0.55 | -0.18±0.32 | | -0.57 | |
| MFTC.ThCtAB | -0.13±0.26 | -0.50 | -0.13±0.23 | -0.56 | -0.04±0.12 | -0.32 | -0.08±0.16 | -0.50 | -0.09±0.19 | | -0.47 | |
| aMT.ThCtAB | -0.02±0.10 | -0.23 | -0.05±0.07 | -0.23 | +0.02±0.08 | +0.31 | -0.01±0.09 | -0.13 | -0.02±0.11 | | -0.21 | |
| ccMF.ThCtAB | -0.13±0.29 | -0.46 | -0.10±0.21 | -0.45 | -0.03±0.14 | -0.19 | -0.09±0.18 | -0.50 | -0.10±0.20 | | -0.48 | |
| Data are mean±standard deviation, in millimetres (mm), except for MFTC.VC and cMF.VC that are shown in cubic millimetres (mm^3^); SRM: standardized response means (mean divided by SD); MFTC.VCtAB: normalized cartilage volume in medial tibia femoral compartment; cMF.VCtAB: normalized cartilage volume in central medial femoral; MFTC.VC: cartilage volume in medial tibia femoral compartment; cMF.VC: volume of cartilage in central medial femoral; cMFTC.ThCtAB: mean cartilage thickness in central medial tibia femoral compartment (weight-bearing); MFTC.ThCtAB: mean cartilage thickness in medial tibia femoral compartment; aMT.ThCtAB: mean cartilage thickness in medial tibia (anterior); ccMF.ThCtAB: mean cartilage thickness in central medial femoral (center); p-values are shown in Table 4 | | | | | | | | | | | |  |
