# Supplementary material for: Mitochondrial DNA (mtDNA) Haplogroups Influence the Progression of Knee Osteoarthritis. Data from the Osteoarthritis Initiative (OAI)
Source: PLoS One. 2014 Nov 12;9(11):e112735. doi: 10.1371/journal.pone.0112735 (PMC4229258; doi:10.1371/journal.pone.0112735)
Supplement: Table S2 — Cross-sectional differences among the mitochondrial DNA (mtDNA) haplogroups in quantitative parameters of cartilage structure collected in an additional and different cohort of (n = 326) knee OA patients in the progression subcohort of the OAI with no follow-up (namely Project 18). (DOCX) [file pone.0112735.s004.docx]

| **Table S2.** Cross-sectional differences among the mitochondrial DNA (mtDNA) haplogroups in quantitative parameters of cartilage structure collected in an additional and different cohort of (n=326) knee OA patients in the progression subcohort of the OAI with no follow-up (namely Project 18) | | | | | | | | | | | | | | | | | | | | | | |  |
| --- | --- | --- | --- | --- | --- | --- | --- | --- | --- | --- | --- | --- | --- | --- | --- | --- | --- | --- | --- | --- | --- | --- | --- |
| **mtDNA haplogroups** | | | | | | | | | | | | | | | | | | | | | | |  |
|  | | | **H (N=125)** | | **J (N=37)** | | | | **T (N=30)** | | | | **Uk (N=75)** | | | | **Others (N=59)** | | | |  | |  |
|  | | | **Mean (SD)** |  | **Mean (SD)** | | **p-value** | | **Mean (SD)** | | **p-value** | | **Mean (SD)** | | **p-value** | | **Mean (SD)** | | **p-value** | |  | |  |
| *Cartilage volume* | | |  |  |  | |  | |  | |  | |  | |  | |  | |  | |  | |  |
| MFTC.VCtAB | 3.37 (0.80) | |  | | 3.26 (0.90) | | 0.529 | | 3.69 (0.93) | | 0.204 | | 3.48 (0.89) | | 0.092 | | 3.25 (0.76) | | 0.984 | |  |  |  |
| MT.VCtAB | 1.63 (0.31) | |  | | 1.57 (0.36) | | 0.306 | | 1.79 (0.35) | | 0.072 | | 1.69 (0.34) | | 0.127 | | 1.59 (0.27) | | 0.927 | |  |  |  |
| cMF.VCtAB | 1.74 (0.53) | |  | | 1.68 (0.58) | | 0.776 | | 1.91 (0.64) | | 0.454 | | 1.80 (0.61) | | 0.120 | | 1.66 (0.53) | | 0.980 | |  |  |  |
| MFTC.VC (x1000) | 3.04 (0.89) | |  | | 2.92 (1.04) | | 0.498 | | 3.57 (1.16) | | 0.034* | | 3.30 (1.06) | | 0.041* | | 2.86 (0.88) | | 0.761 | |  |  |  |
| MT.VC (x1000) | 2.02 (0.58) | |  | | 1.93 (0.67) | | 0.365 | | 2.36 (0.74) | | 0.033* | | 2.19 (0.67) | | 0.058 | | 1.91 (0.55) | | 0.709 | |  |  |  |
| cMF.VC (x1000) | 1.02 (0.37) | |  | | 0.99 (0.41) | | 0.855 | | 1.21 (0.50) | | 0.100 | | 1.10 (0.45) | | 0.067 | | 0.95 (0.37) | | 0.893 | |  |  |  |
| *Cartilage thickness* | | |  |  |  | |  | |  | |  | |  | |  | |  | |  | |  | |  |
| cMFTC.ThCtAB | 4.25 (1.18) | |  | | 4.09 (1.41) | | 0.645 | | 4.76 (1.32) | | 0.133 | | 4.41 (1.39) | | 0.061 | | 4.08 (1.16) | | 0.949 | |  |  |  |
| MFTC.ThCtAB | 3.37 (0.78) | |  | | 3.24 (0.88) | | 0.413 | | 3.69 (0.90) | | 0.191 | | 3.46 (0.88) | | 0.131 | | 3.25 (0.73) | | 0.989 | |  |  |  |
| aMT.ThCtAB | 1.56 (0.33) | |  | | 1.45 (0.33) | | 0.038* | | 1.67 (0.40) | | 0.287 | | 1.55 (0.32) | | 0.640 | | 1.49 (0.32) | | 0.468 | |  |  |  |
| MT.ThCcAB | 1.74 (0.27) | |  | | 1.69 (0.29) | | 0.202 | | 1.88 (0.28) | | 0.038* | | 1.80 (0.29) | | 0.182 | | 1.69 (0.23) | | 0.526 | |  |  |  |
| cMT.ThCtAB | 2.30 (0.55) | |  | | 2.21 (0.70) | | 0.405 | | 2.60 (0.59) | | 0.036* | | 2.39 (0.64) | | 0.125 | | 2.24 (0.50) | | 0.933 | |  |  |  |
| ccMF.ThCtAB | 1.94 (0.73) | |  | | 1.88 (0.79) | | 0.993 | | 2.15 (0.85) | | 0.544 | | 2.02 (0.86) | | 0.081 | | 1.85 (0.75) | | 0.844 | |  |  |  |
| Data are in millimetres (mm), except for MFTC.VC, MT.VC and cMF.VC that are shown in cubic millimetres (mm^3^); SD: standard deviation; MFTC.VCtAB: normalized cartilage volume in medial tibia femoral compartment; MT.VCtAB: normalized cartilage volume in medial tibia; cMF.VCtAB: normalized cartilage volume in central medial femoral; MFTC.VC: cartilage volume in medial tibia femoral compartment; MT.VC: volume of cartilage in medial tibia; cMF.VC: volume of cartilage in central medial femoral; cMFTC.ThCtAB: mean cartilage thickness in central medial tibia femoral compartment (weight bearing); MFTC.ThCtAB: mean cartilage thickness in medial tibia femoral compartment; aMT.ThCtAB: mean cartilage thickness in medial tibia (anterior); MT.ThCcAB: mean cartilage thickness in medial tibia; cMT.ThCtAB: mean cartilage thickness in medial tibia (center); ccMF.ThCtAB: mean cartilage thickness in central medial femoral (center); (#) Multivariate (ANCOVA) analysis adjusting for gender, age, previous surgery, mJSN of the analyzed knee and body mass index (BMI) at baseline, and considering the most common mtDNA haplogroup H as the reference group; (*) indicates statistical significance (p ≤ 0.05) | | | | | | | | | | | | | | | | | | | | | | |  |
